# Retrospective evaluation of nitrofurantoin and pivmecillinam for the treatment of lower urinary tract infections in men

**DOI:** 10.1371/journal.pone.0211098

**Published:** 2019-01-25

**Authors:** Hanna Montelin, Karl-Johan Forsman, Thomas Tängdén

**Affiliations:** Department of Medical Sciences, Section of Infectious Diseases, Uppsala University, Uppsala, Sweden; Northwestern University Feinberg School of Medicine, UNITED STATES

## Abstract

**Objectives:**

This study aimed to retrospectively assess the clinical outcome with nitrofurantoin and pivmecillinam for lower urinary tract infections (UTI) in men. Patients treated with trimethoprim were also included for comparison.

**Methods:**

All prescriptions of the study antibiotics to adult men in Uppsala County, Sweden, during 2012 were extracted. Data on patient characteristics, therapy, clinical outcome and microbiological results were obtained from the electronic medical records. The relative impact of antibiotic therapy, patient factors and pathogens on clinical outcome was assessed with univariate logistic regression using a 95% confidence interval (CI).

**Results:**

832 prescriptions were identified, and 171 patients treated with nitrofurantoin (n = 69), pivmecillinam (n = 57) and trimethoprim (n = 45) met the inclusion criteria. Treatment failure occurred in one patient treated with nitrofurantoin and in four patients treated with pivmecillinam. New prescriptions of UTI antibiotics and relapse within 3 months after completion of therapy were more frequent with nitrofurantoin (34% and 15%) and pivmecillinam (30% and 17%) than trimethoprim (22 and 7%). However, these differences were not statistically significant and substantial heterogeneity was noted between the treatment groups. Urinary tract catheterization was associated with a higher risk for new antibiotic prescriptions (OR 2.34, 95% CI 1.14–4.80; *P* = 0.022) and prostate cancer was associated with a higher incidence of relapse (OR 3.01, 95% CI 1.09–8.29; *P* = 0.042).

**Conclusions:**

The clinical outcome with nitrofurantoin and pivmecillinam was acceptable in comparison with the results of previous studies. These antibiotics are suitable for empirical treatment of lower UTI in men considering their high activity against *Escherichia coli* and limited impact on the intestinal microbiota.

## Introduction

Oral treatment options for urinary tract infections (UTI) are increasingly limited due to emerging resistance and the lack of new antibiotics [1, 2]. Resistance rates to ciprofloxacin and trimethoprim +/- sulphamethoxazole are often ≥ 10% and ≥ 20%, respectively, in the primary pathogen *Escherichia coli* [3–5]. Lower UTI in men usually occurs in patients with structural or neurological abnormalities in the urinary tract. Recurrent infections are frequent because of persistent underlying risk factors for UTI such as urinary tract catheters [6]. Pathogens other than *E*. *coli* are more commonly isolated and resistance levels are generally higher than in other patient groups [2].

As a response to the increasing resistance to standard treatment, old antibiotics that are still effective have been revisited in recent years [7–10]. Nitrofurantoin and pivmecillinam are highly active against *E*. *coli* [1, 2, 11], also against strains producing extended-spectrum beta-lactamases (ESBLs), which may result from their limited impact on the intestinal microbiota and a low risk of resistance development. Clinical data support the use of these antibiotics for uncomplicated cystitis in women [3, 8], while evidence is still insufficient for the treatment of UTI in men.

The increasing resistance rates are considered in the revised guidelines for UTI provided by the Swedish Society of Infectious Diseases published in 2013 [12]. Ciprofloxacin, previously a first-line agent, should be prescribed for lower UTI in men only when there are no other alternatives in order to prevent further resistance development (8% of clinical *E*. *coli* isolates were resistant in 2012 according to national surveillance data) [13]. Trimethoprim (160 mg q12h), is no longer recommended for empirical treatment due to frequent resistance in *E*. *coli* (c. 20%) [13] and should be considered only upon isolation of susceptible bacteria. Instead, nitrofurantoin (50 mg q8h) and pivmecillinam (200 mg q8h), both active against > 90% of *E*. *coli* isolates in Sweden [13], are recommended for empirical treatment despite the lack of clinical evidence for this indication. The recommended treatment duration for lower UTI in men is 7 days [12].

In this study, we describe patient characteristics, medical conditions, causative pathogens, treatment regimens and clinical outcome in male patients prescribed nitrofurantoin and pivmecillinam for lower UTI in Uppsala County, Sweden, during 2012. Data on patient characteristics, treatment, microbiological results and outcome were extracted through retrospective review of the electronic medical records and were obtained also for patients treated with trimethoprim during the same period to allow a comparison between the three drugs.

## Materials and methods

### Study design and eligibility criteria

All prescriptions of nitrofurantoin, pivmecillinam and trimethoprim to adult men during 2012 were identified through the electronic medical records database of Uppsala County. Data on patient characteristics, antibiotic treatment, clinical outcome and microbiological results within a 3-month follow-up period were extracted. The study was approved by the Regional Ethical Review Board of Uppsala and was conducted in accordance with the Declaration of Helsinki. All data were analysed anonymously and informed consent was waived due to the retrospective observational design of the study.

Inclusion criteria were (1) age ≥18 years, (2) at least one of the following conditions: dysuria, frequency or urgency of micturition, suprapubic pain, urinary retention or haematuria, (3) significant (i.e. ≥10^3^ colony forming units, cfu/mL) pure culture growth of bacteria in urine and (4) susceptibility of the isolated bacteria to the prescribed antibiotic. Exclusion criteria were (1) concurrent therapy with other systemic antibiotics, (2) suspected prostatitis, (3) other indication than UTI, (4) antibiotics intended as prophylaxis, (5) death during follow up, (6) incomplete or lacking medical records and (7) fever > 38°C after onset of symptoms. Patients with prostatitis were not included because they usually receive trimethoprim for 3–12 weeks, which is much longer than the normal treatment duration for lower UTI (7 days).

### Outcome definitions

Treatment failure was defined as prescription of another antibiotic during the intended treatment period due to clinical deterioration or persistent symptoms. Relapse was defined as all of the following: (1) a new prescription of antibiotics within 3 months after completion of therapy, (2) recurrent urinary tract symptoms and (3) isolation of the same bacterial species with a similar antibiotic susceptibility profile as in the first episode.

### Bacteria and antibiotic susceptibilities

Microbiological results as reported from the Department of Clinical Microbiology at Uppsala University Hospital were extracted from the medical records. Bacterial cultures and species determination were performed using either conventional methods or a Vitek 2 instrument (bioMérieux, Marcy-I´Etoile, France). Antibiotic susceptibilities were determined with disk diffusion or the gradient method (Etest, bioMérieux, Marcy-I´Etoile, France) and interpreted according to EUCAST definitions [14]. Susceptibility to mecillinam in *Enterobacter sp*., *Citrobacter sp*. and *Serratia sp*. was tested and interpreted as recommended for *E*. *coli* in accordance with local guidelines during the study period.

### Statistical analysis

Patient characteristics are described with median (range) for numerical variables and with numbers and percentage for categorical variables. Outcome in relation to antibiotics, duration of therapy, potential risk factors and Gram-negative vs. Gram-positive bacteria was assessed with univariate logistic regression. The results are presented with odds ratios (OR) and 95% confidence intervals (CI). The association of relapse and duration of treatment was explored with antibiotic type as an additional covariate with a log likelihood ratio test comparing the full model with an interaction term (antibiotic type and duration) and the smaller model without the interaction term. Subgroup analyses to evaluate the risk of relapse with different treatment durations (≤ 7 vs. > 7 days) were performed using Fisher´s exact test. Statistical significance was set at *P*< 0.05 (two-sided). The statistical analysis was performed using R version 3.3.2 (R Foundation for Statistical Computing, Vienna, Austria).

## Results

### Patients

**Fig 1:**
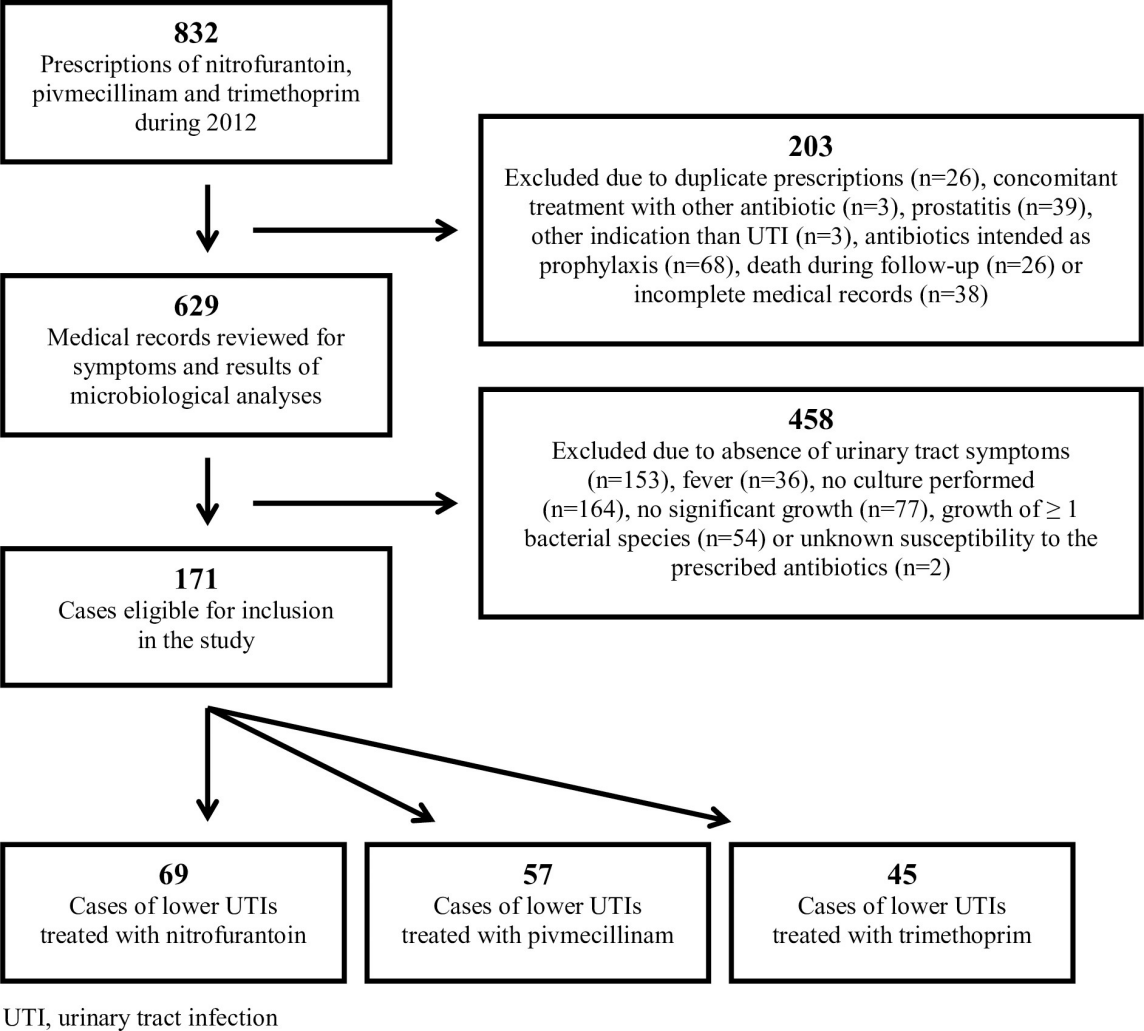
Flow chart of the inclusion process.

During 2012, 832 prescriptions of nitrofurantoin, pivmecillinam and trimethoprim were made to adult male patients in Uppsala County. 171 patients were eligible for inclusion in the analysis. The most common reasons for exclusion were a lack of documented urinary tract symptoms (n = 153) or urinary cultures (n = 164), as shown in Fig 1. Death during follow-up occurred in 26 cases. None of the patients were considered to have died from UTI or adverse events related to the antibiotic treatment. Most patients were treated in primary care (93%) (Table 1). The median age was 70 years with small observed differences between the treatment groups. Dysuria was the most frequently reported symptom (61%) followed by urgency of micturition (51%). Half of the patients had received antibiotics for a suspected UTI during the preceding 3 months. Potential risk factors such as underlying medical conditions or urinary catheters were present in 69% of the cases (Table 1).

**Table 1:** Patient characteristics, symptoms, recent antibiotic therapy, risk factors for urinary tract infections and dosage regimens.

Variable	No. (%)			
	Nitrofurantoin n = 69	Pivmecillinam n = 57	Trimethoprim n = 45	Total n = 171
Age, years, median (range)	69 (21–93)	67 (19–91)	71 (21–90)	70 (19–93)
Prescription site				
Primary care	66 (96)	54 (95)	39 (87)	159 (93)
Hospital	2 (3)	3 (5)	3 (7)	8 (5)
Nursing home	1 (1)	0 (0)	3 (7)	4 (2)
Symptoms				
Dysuria	42 (61)	35 (61)	28 (62)	105 (61)
Urinary urgency	40 (58)	22 (39)	26 (58)	88 (51)
Increased frequency of urination	11 (16)	12 (21)	7 (16)	30 (18)
Macroscopic hematuria	5 (7)	3 (5)	3 (7)	11 (6)
Recent UTI therapy	47 (68)	24 (52)	16 (36)	87 (51)
Risk factors	49 (71)	41 (72)	28 (62)	118 (69)
Urinary tract catheterization (any)	20 (29)	15 (26)	10 (22)	45 (26)
Intermittent catheterization	16 (23)	12 (21)	8 (18)	36 (21)
Indwelling urethral catheter	3 (4)	3 (5)	2 (4)	8 (5)
Indwelling supra-pubic catheter	1 (1)	0 (0)	0 (0)	1 (1)
Benign prostate hypertrophy	21 (30)	14 (25)	5 (11)	40 (23)
Prostate cancer	9 (13)	9 (16)	9 (20)	27 (16)
Diabetes mellitus	1 (1)	6 (11)	10 (22)	25 (15)
Neurogenic bladder dysfunction	7 (10)	7 (12)	3 (7)	17 (10)
Bladder cancer	4 (6)	3 (5)	1 (2)	8 (5)
Urinary tract anomaly	2 (3)	5 (9)	2 (4)	9 (5)
Urethral stone	4 (6)	1 (2)	0 (0)	5 (3)
Recent urological procedure	2 (3)	2 (4)	0 (0)	4 (2)
Immunosuppression	1 (1)	3 (5)	0 (0)	4 (2)
Renal transplant	0 (0)	1 (2)	0 (0)	1 (1)
Dosage, no. (%)	50 mg q8h, 65 (94)	200 mg q8h, 37 (65)	160 mg q12h, 44 (98)	
	50 mg q12h, 2 (3)	200 mg q12h, 17 (30)	300 mg q24h, 1 (2)	
	100 mg q8h, 2 (3)	400 mg q8h, 2 (4)		
		400 mg q12h, 2 (4)		

UTI, urinary tract infection

### Bacteria and antibiotic susceptibility

Notable differences existed in causative bacteria between the treatment groups (Table 2). Most isolates were Gram-negative bacteria belonging to the Enterobacteriaceae family (64%). *E*. *coli* was the most frequent pathogen (49%), followed by *Enterococcus faecalis* (23%), which was predominant in patients treated with nitrofurantoin. More than 95% of *E*. *coli* isolates were susceptible to nitrofurantoin and pivmecillinam, whereas 31% were resistant to ciprofloxacin and trimethoprim (Table 3). Of the *E*. *coli* isolates, 12 (14%) were reported to produce ESBLs.

**Table 2:** Pathogens detected in urinary cultures.

Pathogen	No. (%)			
	Nitrofurantoin n = 69	Pivmecillinam n = 57	Trimethoprim n = 45	Total n = 171
**Gram-negative bacteria**	23 (34)	57 (100)	29 (64)	109 (64)
*Escherichia coli*	23 (34)	41 (72)	20 (44)	84 (49)
*Proteus spp*.		9 (16)	2 (4)	11 (6)
*Klebsiella spp*.		4 (7)	2 (4)	6 (4)
*Enterobacter spp*.		1 (2)	3 (7)	4 (2)
*Providencia sp*.			2 (4)	2 (1)
*Citrobacter sp*.		1 (2)		1 (1)
*Serratia sp*.		1 (2)		1 (1)
**Gram-positive bacteria**	46 (67)	0 (0)	16 (36)	62 (36)
*Enterococcus faecalis*	40 (58)			40 (23)
*Coagulase-negative staphylococci*			9 (20)	9 (5)
*Staphylococcus aureus*			7 (16)	7 (4)
*Streptococcus agalactiae*	4 (6)			4 (2)
*Staphylococcus saprophyticus*	2 (4)			2 (1)

**Table 3:** Antibiotic susceptibilities of detected pathogens.

Pathogen	No. (%) susceptible					
	AMP	CFR	CIP	MEC	NIT	TMP
**Gram-negative bacteria**						
*Escherichia coli*	61 (73)	70 (83)	58 (69)	82 (98)	80 (96)	58 (69)
*Proteus spp*.	7 (64)	7 (64)	11 (100)	11 (100)	0 (0)	7 (64)
*Klebsiella spp*.	0 (0)	6 (100)	6 (100)	6 (100)	0 (0)	5 (83)
*Enterobacter spp*.	0 (0)	0 (0)	4 (100)	4 (100)^a^	0 (0)	4 (100)
*Providencia sp*.	0 (0)	0 (0)	1 (50)	0 (0)	0 (0)	2 (100)
*Citrobacter sp*.	0 (0)	0 (0)	1 (100)	1 (100)^a^	0 (0)	1 (100)
*Serratia sp*.	0 (0)	0 (0)	1 (100)	1 (100)^a^	0 (0)	1 (100)
**Gram-positive bacteria**						
*Enterococcus faecalis*	38 (97)	0 (0)	0 (0)	0 (0)	39 (100)	ND
*Coagulase-negative staphylococci*	ND	ND	ND	ND	ND	9 (100)
*Staphylococcus aureus*	ND	7 (100)	ND	ND	ND	7 (100)
*Streptococcus agalactiae*	4 (100)	ND	ND	ND	4 (100)	ND
*Staphylococcus saprophyticus*	2 (100)	2 (100)	ND	ND	2 (100)	2 (100)

AMP, ampicillin; CFR, cefadroxil; CIP, ciprofloxacin; MEC, mecillinam; NIT, nitrofurantoin; TMP, trimethoprim; ND, not determined

^a^ Susceptibility to mecillinam was tested and interpreted as recommended for *E*. *coli*.

### Antibiotic treatment

The median duration of therapy was 7 days in the nitrofurantoin and pivmecillinam groups, in accordance with current guidelines, and 10 days for trimethoprim (Table 4). Five days of therapy was used in 38% and 21% of patients treated with nitrofurantoin and pivmecillinam, respectively. In the trimethoprim group, only three patients received less than 7 days of therapy; two patients were treated for 5 days and one patient for 3 days. Dosing regimens were in most cases adherent to the recommendations: 50 mg q8h for nitrofurantoin, 200 mg q8h for pivmecillinam and 160 mg q12h for trimethoprim (Table 1). However, almost a third of the patients treated with pivmecillinam were prescribed a lower dosage of 200 mg q12h.

**Table 4:** Details on duration of therapy and outcome of all patients and subgroups.

Variable	Nitrofurantoin n = 69	Pivmecillinam n = 57	Trimethoprim n = 45	Total n = 171
**Duration of therapy, days, median (range)**				
All patients	7 (5–21)	7 (5–14)	10 (3–14)	7 (3–21)
Relapse	10 (5–21)	7 (5–7)	10 (7–14)	7 (5–21)
No relapse	7 (5–21)	7 (5–14)	10 (3–14)	7 (3–14)
**Treatment failure, no. (%)**				
All patients (n = 171)	1 of 69 (1)	4 of 57 (7)	0 of 45 (0)	5 (3)
No recent UTI therapy (n = 84)	0 of 22 (0)	1 of 33 (3)	0 of 29 (0)	1 (1)
Recent UTI therapy (n = 87)	1 of 47 (2)	3 of 24 (13)	0 of 16 (0)	4 (5)
Gram-negative bacteria (n = 109)	0 of 23 (0)	4 of 57 (7)	0 of 29 (0)	4 (4)
Gram-positive bacteria (n = 62)	1 of 46 (2)	-	0 of 16 (0)	1 (2)
No risk factors (n = 53)	0 of 20 (0)	1 of 16 (6)	0 of 17 (0)	1 (2)
Any risk factor (n = 118)	1 of 49 (2)	3 of 41 (7)	0 of 28 (0)	4 (3)
Catheterization (n = 45)	0 of 20 (0)	2 of 15 (13)	0 of 10 (0)	2 (4)
Other risk factors (n = 73)	1 of 29 (3)	1 of 26 (4)	0 of 18 (0)	2 (3)
**New prescription, no (%)**				
All patients (n = 166)	23 of 68 (34)	16 of 53 (30)	10 of 45 (22)	49 (30)
No recent UTI therapy (n = 83)	6 of 22 (27)	9 of 32 (28)	10 of 29 (34)	25 (30)
ecent UTI therapy (n = 83)	17 of 46 (37)	6 of 21 (29)	0 of 16 (0)	23 (28)
Gram-negative bacteria (n = 106)	10 of 23 (43)	16 of 53 (30)	8 of 29 (28)	34 (32)
Gram-positive bacteria (n = 61)	11 of 45 (24)	-	2 of 16 (13)	13 (21)
Treatment duration ≤ 7 days (n = 100)	14 of 45 (31)	13 of 35 (37)	3 of 20 (15)	30 (30)
Treatment duration > 7 days (n = 66)	9 of 23 (39)	3 of 18 (17)	7 of 25 (28)	19 (29)
No risk factors (n = 52)	4 of 20 (20)	3 of 15 (20)	3 of 17 (18)	10 (19)
Any risk factor (n = 114)	19 of 48 (40)	12 of 38 (32)	7 of 28 (25)	37 (32)
Catheterization (n = 43)	10 of 20 (50)	5 of 13 (38)	4 of 10 (40)	19 (44)
Other risk factors (n = 71)	9 of 28 (32)	7 of 25 (28)	3 of 18 (17)	18 (25)
**Time to new prescription, days, median (range)**	14 (1–84)	15.5 (2–84)	14 (1–91)	14 (1–91)
**Relapse, no. (%)**				
All patients (n = 166)	10 of 68 (15)	9 of 53 (17)	3 of 45 (7)	22 (13)
No recent antibiotic therapy (n = 83)	2 of 22 (9)	7 of 32 (22)	3 of 29 (10)	12 (14)
Recent antibiotic therapy (n = 83)	8 of 46 (17)	2 of 24 (10)	0 of 16 (0)	10 (12)
Gram-negative bacteria (n = 106)	4 of 23 (17)	9 of 53 (17)	2 of 29 (7)	15 (14)
Gram-positive bacteria (n = 61)	6 of 45 (13)	-	1 of 16 (6)	7 (11)
Treatment duration ≤ 7 days (n = 100)	5 of 45 (11)	9 of 35 (26)	1 of 20 (5)	15 (15)
Treatment duration > 7 days (n = 66)	5 of 23 (22)	0 of 18 (0)	2 of 25 (8)	7 (11)
No risk factors (n = 52)	2 of 20 (10)	3 of 15 (20)	1 of 17 (6)	6 (12)
Risk factors (n = 114)	8 of 48 (17)	6 of 38 (16)	2 of 28 (7)	16 (14)
Catheterization (n = 43)	4 of 20 (20)	3 of 13 (23)	1 of 10 (10)	8 (19)
Other risk factors (n = 71)	4 of 28 (14)	3 of 25 (12)	1 of 18 (6)	8 (11)
**Time to relapse, days, median (range)**	14 (10–84)	10 (2–42)	10 (10–14)	10 (2–84)

UTI, urinary tract infection

### Treatment failure

Treatment failure was observed in one patient with diabetes mellitus treated with nitrofurantoin (Table 4) who had persistent symptoms after 5 days with no additional culture performed. Also, four patients treated with pivmecillinam were switched to other antibiotics after 1, 3, 4 and 6 days of therapy because of high fever or persistent symptoms. Repeated urinary sampling was performed in one of these patients and showed no bacterial growth. The patient who developed fever had no recorded risk factors for UTIs while urinary tract catheters were present in two of the patients. Four of the five patients with treatment failure had recent UTI therapy the preceding 3 months. No treatment failure was noted in the trimethoprim group.

### New prescriptions

New antibiotic prescriptions for suspected recurrent UTI were made in 49 of the patients (Tables 4 and 5). Two patients who were prescribed antibiotics were excluded from the analysis; one patient was prescribed ciprofloxacin for a subcutaneous abscess and one patient received pivmecillinam as long-term prophylaxis for UTI. The median interval between completion of treatment and a new prescription was 14 days (range 1–91 days). New prescriptions were more frequently observed with nitrofurantoin (34%, OR 1.75, 95% CI 0.74–4.15; *P* = 0.204) and pivmecillinam (30%, OR 1.37, 95% CI 0.55–3.39, *P* = 0.502) than with trimethoprim (22%). However, these differences were not statistically significant. Treatment duration > 7 days was not associated with a reduced risk (OR 1.10, 95% CI 0.56–2.16, *P* = 0.780). A higher incidence of new prescriptions was noted in patients with urinary tract catheters (OR 2.34, 95% CI 1.14–4.80; *P* = 0.022) (Table 5).

**Table 5:** Univariate analysis for risk factors of new prescription and relapse.

	New prescription			Relapse		
	OR	95% CI	*P*	OR	95% CI	*P*
Antibiotics						
Trimethoprim	ref			ref		
Nitrofurantoin	1.75	0.74–4.15	0.204	2.37	0.62–9.15	0.209
Pivmecillinam	1.37	0.55–3.39	0.502	2.62	0.67–10.34	0.168
Recent UTI therapy	0.90	0.46–1.75	0.753	0.78	0.32–1.91	0.586
Gram-positive bacteria	0.54	0.26–1.12	0.089	0.80	0.31–2.08	0.640
Treatment duration > 7 d	1.10	0.56–2.16	0.780	0.87	0.34–2.21	0.771
Any risk factors	2.12	0.97–4.67	0.052	1.23	0.45–3.34	0.683
Urinary tract catheterization	2.34	1.14–4.80	0.022	1.73	0.67–4.45	0.265
Benign prostate hypertrophy	1.71	0.08–3.62	0.165	1.27	0.46–3.49	0.650
Prostate cancer	1.92	0.82–4.50	0.141	3.01	1.09–8.29	0.042
Diabetes mellitus	1.21	0.48–3.01	0.692	0.91	0.25–3.34	0.888
Neurogenic bladder disorder	1.04	0.35–3.13	0.942	0.40	0.05–3.14	0.322

OR, odds ratio; CI, confidence interval; ref, reference; UTI, urinary tract infection

### Relapse

Relapse was noted in 13% of the patients (Tables 4 and 5), in most cases (68%) within 14 days, and was more frequently observed in patients treated with nitrofurantoin (15%, OR 2.37, 95% CI 0.62–9.15, *P* = 0.209) or pivmecillinam (17%, OR 2.62, 95% CI 0.67–10.34, *P* = 0.168) than with trimethoprim (7%). These differences were not statistically significant. Overall, duration of therapy > 7 days did not result in a lower risk for relapse. However, in the subgroup of patients treated with pivmecillinam a longer treatment duration was associated with a significantly lower risk; none of the 18 patients treated for more than 7 days relapsed (17 patients were treated for 10 days and one patient for 14 days), as compared to nine of 35 (26%) patients treated for ≤ 7 days (*P* = 0.045). No association was found between the dosage of pivmecillinam and relapse (16% with 200 mg q8h and 12% with 200 mg q12h). Prostate cancer was associated with a significantly higher risk for relapse (OR 3.01, 95% CI 1.09–8.29; *P* = 0.042) (Table 5).

### Outcome in relation to bacterial species

Relapse occurred in 13 of 84 (15%) infections caused by *E*. *coli*; four of 23 (17%) of patients treated with nitrofurantoin, eight of 41 (20%) of patients treated with pivmecillinam and one of 20 (5%) patients treated with trimethoprim. In patients infected by ESBL-producing *E*. *coli* one of nine patients treated with nitrofurantoin relapsed and treatment failure or relapse were recorded in two of three patients treated with pivmecillinam. All 40 patients infected by *E*. *faecalis* were prescribed nitrofurantoin and relapse was noted in six cases (15%). No subgroup analysis was performed for other bacterial species due to the low number of cases.

## Discussion

In this study, prescriptions of nitrofurantoin, pivmecillinam and trimethoprim were reviewed retrospectively using strict criteria for inclusion and outcome. As expected, older age, recent antibiotic treatment and presence of urinary tract abnormalities and catheters were frequent in the study group. The causative bacteria were also in line with previous reports [2, 15] but with a relatively large proportion of *E*. *faecalis* probably due to the selection of patients prescribed nitrofurantoin. Resistance to ciprofloxacin and trimethoprim in *E*. *coli* was 31%, which is higher than reported national data (8% and 20%, respectively) [13]. This finding may in part be explained by the fact that nitrofurantoin and pivmecillinam are more likely to be used in cases where resistance is reported for other antibiotics. Furthermore, multiple risk factors for resistance were present in the study patients, e.g. older age, urogenital comorbidities and recent antibiotic treatment. Still, susceptibility rates > 95% in *E*. *coli* were noted for nitrofurantoin and pivmecillinam.

The overall clinical response was comparable to previous studies considering that risk factors for recurrent infections were present in most patients. Treatment failure was noted in 3%, a new antibiotic prescription in 30% and relapse with repeated bacterial growth within 3 months in 13% of the patients. In a recent study on the efficacy and safety of nitrofurantoin in male patients with lower UTIs, clinical cure was reported in 77% of the cases [10]. Still, limited data exist for this indication and the comparison between studies is complicated by differences in methodologies and patients. For example, in the study of Ingalsbe et al. [10], fever and flank pain were used as inclusion criteria and the follow-up period was only 14 days. Dysuria, which was associated with worse outcome, was present in only 21% of the patients [10] as compared to 61% in our material.

A large proportion of patients did not meet the inclusion criteria resulting in a small sample size in each treatment group included in the analysis. Inappropriate antibiotic prescriptions based on isolation of bacteria in urine despite a lack of urinary tract symptoms (i.e., asymptomatic bacteriuria) were frequent. Urinary cultures, which are always recommended in these patients according to national treatment guidelines because of the high risk for resistant bacteria, were performed in only 71% of the cases. In this study, the presence of a urinary tract catheter and prostate cancer were the only statistically significant risk factors for new antibiotic prescriptions and relapse. Our results further support the recommendation to remove urinary tract catheters when possible to reduce the risk for UTI [6]. Screening and treatment for asymptomatic bacteriuria should be avoided to prevent unnecessary antibiotic use. Also, careful clinical assessment is important to distinguish cystitis from conditions engaging the prostatic gland, where antibiotics with adequate tissue penetration are required to prevent progression to severe systemic infections.

This study has several limitations, including the small sample size, multiple testing, the retrospective study design associated with a risk of bias in the selection of antibiotic therapy and differences between the treatment groups. The great variation in bacterial species between the groups is an important limitation, which may have influenced clinical outcome. A shorter duration of therapy was noted for patients treated with nitrofurantoin or pivmecillinam, which may have increased the risks of treatment failure and relapse. Several potential risk factors that were unevenly distributed between treatment arms (less frequent in the trimethoprim group) may also have affected clinical outcome although they were not found statistically significant in this study. Because of the low number of events, possible confounders could not be explored. Symptoms, bacterial growth and relapse rates may have been underestimated and some infections misclassified as lower UTI rather than prostatitis or pyelonephritis because of the lack of standardised data collection and follow up. Furthermore, new prescriptions of UTI antibiotics during follow up, used as an indicator of suspected relapse, may in some cases have reflected recurrent infections, other indications for antibiotic therapy or unnecessary treatment for asymptomatic bacteriuria.

Therefore, our results should be interpreted with caution and the results regarding treatment regimens are not conclusive. Still, due to the paucity of other data we believe that our study provides new important information of interest in the management of these patients. The strict criteria for inclusion and relapse and the consecutive inclusion of all patients in the region based on reliable data on individual prescriptions, are strengths of this study in relation to many previous publications on lower UTI in men.

In absolute numbers, clinical outcome was worse in the nitrofurantoin and pivmecillinam groups. Use of these antibiotics has previously been discouraged for the treatment of UTIs in men because of their pharmacokinetic properties resulting in low antibiotic concentrations in prostate [16]. In this study, no association was found between dosage and outcome. However, higher dosage (e.g., 100 mg q8h of nitrofurantoin and 400 mg q8h of pivmecillinam) are used elsewhere [10, 17] and may be considered in this patient group to increase tissue concentrations and possibly improve outcome. As for treatment duration, 7 to 14 days are usually advocated for lower UTI in men and catheter-related infections [4–6] although clinical evidence to support these recommendations is lacking. In this study, the duration of treatment was longer in the trimethoprim group, which may have contributed to the lower risk of relapse in these patients. With pivmecillinam, 26% of patients treated for ≤ 7 days relapsed and none of those who received 10 or 14 days of therapy. Thus, a longer duration of therapy could potentially be of benefit to these patients and warrants further investigation.

## Conclusion

We conclude that nitrofurantoin and pivmecillinam are suitable for empirical treatment of lower UTI in men considering their high activity against *E*. *coli* and favourable ecological profiles, the high resistance levels to trimethoprim and the need to preserve quinolones and other key antibiotics for more severe infections. However, individual assessment of patient-specific risk factors for resistant bacteria is always required. Prospective randomized studies with a larger sample size are needed to establish the potential differences in efficacy between antibiotics, dosage regimens and treatment durations for these infections.
